# Long-term outcomes of patients with large B-cell lymphoma treated with axicabtagene ciloleucel and prophylactic corticosteroids

**DOI:** 10.1038/s41409-023-02169-z

**Published:** 2024-01-04

**Authors:** Olalekan O. Oluwole, Edouard Forcade, Javier Muñoz, Sophie de Guibert, Julie M. Vose, Nancy L. Bartlett, Yi Lin, Abhinav Deol, Peter McSweeney, Andre H. Goy, Marie José Kersten, Caron A. Jacobson, Umar Farooq, Monique C. Minnema, Catherine Thieblemont, John M. Timmerman, Patrick Stiff, Irit Avivi, Dimitrios Tzachanis, Yan Zheng, Saran Vardhanabhuti, Jenny Nater, Rhine R. Shen, Harry Miao, Jenny J. Kim, Tom van Meerten

**Affiliations:** 1 Vanderbilt University Medical Cancer Center, Nashville, TN USA; 2 Service d’Hématologie Clinique et Thérapie Cellulaire, Centre Hospitalier Universitaire de Bordeaux, F-33000 Bordeaux, France; 3 Banner MD Anderson Cancer Center, Gilbert, AZ USA; 4 Hématologie Clinique, Centre Hospitalier Universitaire de Rennes, Rennes, France; 5 University of Nebraska Medical Center, Omaha, NE USA; 6 Washington University School of Medicine and Siteman Cancer Center, St Louis, MO USA; 7 Mayo Clinic, Rochester, MN USA; 8 Karmanos Cancer Center, Wayne State University, Detroit, MI USA; 9 Colorado Blood Cancer Institute, Denver, CO USA; 10 John Theurer Cancer Center, Hackensack, NJ USA; 11 Amsterdam UMC, Location University of Amsterdam, Cancer Center Amsterdam, Amsterdam (on behalf of HOVON/LLPC), The Netherlands; 12 Dana-Farber Cancer Institute, Boston, MA USA; 13 University of Iowa, Iowa City, IA USA; 14 University Medical Center Utrecht (on behalf of HOVON/LLPC), Utrecht, The Netherlands; 15 Paris University, Assistance publique-Hôpitaux de Paris, Hemato-oncology, F-75010 Paris, France; 16 University of California, Los Angeles, David Geffen School of Medicine, Los Angeles, CA USA; 17 Loyola University Chicago Stritch School of Medicine, Maywood, IL USA; 18 Tel Aviv Sourasky Medical Center and Sackler Faculty of Medicine, Tel Aviv University, Tel Aviv, Israel; 19 University of California San Diego, La Jolla, CA USA; 20 Kite, a Gilead Company, Santa Monica, CA USA; 21 University Medical Center Groningen, Groningen (on behalf of HOVON/LLPC), The Netherlands

**Keywords:** B-cell lymphoma, Drug development, Adverse effects

## Abstract

ZUMA-1 safety management cohort 6 investigated the impact of prophylactic corticosteroids and earlier corticosteroids and/or tocilizumab on the incidence and severity of cytokine release syndrome (CRS) and neurologic events (NEs) following axicabtagene ciloleucel (axi-cel) in patients with relapsed/refractory large B-cell lymphoma (R/R LBCL). Prior analyses of cohort 6 with limited follow-up demonstrated no Grade ≥3 CRS, a low rate of NEs, and high response rates, without negatively impacting axi-cel pharmacokinetics. Herein, long-term outcomes of cohort 6 (*N* = 40) are reported (median follow-up, 26.9 months). Since the 1-year analysis (Oluwole, et al*.* *Blood*. 2022;138[suppl 1]:2832), no new CRS was reported. Two new NEs occurred in two patients (Grade 2 dementia unrelated to axi-cel; Grade 5 axi-cel–related leukoencephalopathy). Six new infections and eight deaths (five progressive disease; one leukoencephalopathy; two COVID-19) occurred. Objective and complete response rates remained at 95% and 80%, respectively. Median duration of response and progression-free survival were reached at 25.9 and 26.8 months, respectively. Median overall survival has not yet been reached. Eighteen patients (45%) remained in ongoing response at data cutoff. With ≥2 years of follow-up, prophylactic corticosteroids and earlier corticosteroids and/or tocilizumab continued to demonstrate CRS improvement without compromising efficacy outcomes, which remained high and durable.

## Introduction

Axicabtagene ciloleucel (axi-cel), an autologous anti-CD19 chimeric antigen receptor (CAR) T-cell therapy, was approved for relapsed/refractory large B-cell lymphoma (R/R LBLC) after ≥2 lines of systemic therapy based on the ZUMA-1 registrational study in refractory LBCL (NCT02348216) [1–3]. ZUMA-1 pivotal cohorts 1 + 2 (*N* = 101) demonstrated high, durable responses (83% objective response rate [ORR]; 58% complete response [CR] rate), and a manageable safety profile with long-term follow-up (median, 27.1 months) [4]. Grade ≥3 cytokine release syndrome (CRS) and neurologic events (NEs) were reported in 11% and 31% of patients, respectively. With 63.1-months median follow-up, median overall survival (OS) was 25.8 months (95% CI, 12.8–not estimable) and the 5-year OS rate was 43% (95% CI, 33–52%) [5].

The clinical promise of CAR T-cell therapy is challenged by CRS and NEs, which are acute toxicities that can be life-threatening, requiring careful management and monitoring [6, 7]. Therefore, CRS and NE management have been evaluated to optimize safety outcomes without compromising efficacy [6, 7], and several exploratory safety management cohorts were added to ZUMA-1 [8–10]. Cohort 6 evaluated the impact of prophylactic corticosteroids and earlier corticosteroid and/or tocilizumab intervention on the incidence and severity of CRS and NEs [10]. With 14.9-months median follow-up, cohort 6 demonstrated lower rates of Grade ≥3 CRS and NEs (no Grade ≥3 CRS; 15% Grade ≥3 NEs) than cohorts 1 + 2, and high, durable response rates (95% ORR, 80% CR, and 53% ongoing responses) [11]. Here, long-term data from ZUMA-1 cohort 6 are reported with at least 2 years of follow-up for all patients, including a competing risk analysis of OS performed after propensity score matching (PSM) of patients in cohort 6 and cohorts 1 + 2.

## Methods

### Patients and study design

Full study procedures for ZUMA-1 cohort 6 were previously reported [10]. Patient eligibility and additional study design details are noted within the Supplemental methods. Patients in cohort 6 received conditioning chemotherapy for 3 days (cyclophosphamide 500 mg/m^2^/day and fludarabine 30 mg/m^2^/day on days –5, –4, and –3) prior to a single intravenous infusion of axi-cel (target dose, 2 × 10^6^ CAR T cells/kg) on day 0. Patients received once-daily corticosteroid prophylaxis (oral dexamethasone 10 mg) on days 0 (before axi-cel), 1, and 2, and earlier corticosteroids and/or tocilizumab for CRS and NE management (Fig. S1). Unlike cohorts 1 + 2, patients in cohort 6 could receive optional bridging therapy after leukapheresis at the investigator’s discretion [10].

### Endpoints and assessments

The primary endpoints were incidence and severity of CRS and NEs, which were identified and graded as previously reported [10]. Briefly, severity of CRS was graded per modified Lee 2014 criteria [12]. NEs were identified using a Medical Dictionary for Regulatory Activities version 24.1 search term list that was developed based on a modification of the specific search strategy by Topp et al [13], with severity graded per National Cancer Institute Common Terminology Criteria for Adverse Events version 4.03. Secondary endpoints included adverse event (AE) incidence (Supplemental methods), ORR (partial response [PR] or CR as assessed by investigator per revised International Working Group Response Criteria for Malignant Lymphoma) [14], duration of response (DOR), progression-free survival (PFS), OS, and CAR T-cell levels in blood. The cumulative incidence of non–lymphoma-related mortality was assessed in a post hoc analysis. The associations between pharmacokinetic parameters (median peak CAR T-cell levels and area under the curve within the first 28 days after treatment [AUC_0-28_]) and severity of CRS and NEs were also examined.

### Statistical analysis

For cohort 6, all endpoints were analyzed descriptively; no formal hypothesis was tested [10]. Disease assessment after initiation of new anticancer therapy (excluding stem cell transplant) was not included in DOR or PFS derivations. Descriptive *P* values, calculated by Wilcoxon 2-sample test, were generated to compare pharmacokinetic parameters with toxicity severity. As previously reported [10], an exploratory PSM analysis [15, 16] was performed to retrospectively compare outcomes for patients in cohort 6 and cohorts 1 + 2 (Supplemental methods). Matched cohorts were identified after balancing for the following key baseline disease characteristics: tumor burden, International Prognostic Index score, number of prior lines of chemotherapy, disease stage, and lactate dehydrogenase level. Patients were selected using 1:1 nearest neighbor propensity score matching and caliper option. The cumulative incidence function was compared between competing risks of lymphoma-related and non–lymphoma-related deaths for OS on matched patients. Additional statistical methods can be found in the **Supplement**.

## Results

### Patients

Forty-two patients were enrolled and leukapheresed; 40 received conditioning chemotherapy and axi-cel treatment [10]. Of 21 patients (53%) who received bridging therapy, the most common regimens (used in three or more patients) were corticosteroids (9 [23%]); rituximab with bendamustine and corticosteroids (4 [10%]); and rituximab with bendamustine (3 [8%]) [10]. As of 16 December 2021, median follow-up was 26.9 months (range, 24.0–30.1). Patient and disease characteristics at baseline were previously reported [10].

### Safety

All 40 patients reported Grade ≥3 treatment-emergent AEs (TEAEs; Table 1). The most common Grade ≥3 TEAEs were neutropenia (80%), leukopenia (40%), and thrombocytopenia (28%). Serious any-grade TEAEs occurred in 24 patients (60%), and 20 patients (50%) reported Grade ≥3 events (Table S1). Prolonged Grade ≥3 cytopenias (i.e., those present on or after 30 days from axi-cel infusion) were reported in 21 patients (53%) (Table S2).

**Table 1:** Summary of treatment-emergent adverse events since start of study.

*n* (%)	Any grade	Grade ≥ 3
Any	40 (100)	40 (100)
Pyrexia	34 (85)	5 (13)
Neutropenia^a^	33 (83)	32 (80)
Hypotension	22 (55)	5 (13)
Fatigue	18 (45)	1 (3)
Leukopenia^b^	17 (43)	16 (40)
Thrombocytopenia^c^	16 (40)	11 (28)
Confusional state	15 (38)	1 (3)
Constipation	15 (38)	0
Nausea	14 (35)	1 (3)
Anemia	13 (33)	8 (20)
Headache	13 (33)	0
Diarrhea	11 (28)	1 (3)
Hypokalemia	11 (28)	2 (5)
Hypophosphatemia	11 (28)	6 (15)
Arthralgia	9 (23)	0
Tremor	9 (23)	1 (3)
Chills	8 (20)	0
Decreased appetite	8 (20)	0
Dyspnea	8 (20)	2 (5)
Hypogammaglobulinemia	8 (20)	0
Hypoxia	8 (20)	3 (8)
Vomiting	8 (20)	1 (3)

Shown are treatment-emergent adverse events of any grade occurring in ≥20% of patients, and worst Grade 3 or 4 events occurring in ≥10% of patients. Adverse events were coded using Medical Dictionary for Regulatory Activities version 24.1; severity was graded using the National Cancer Institute Common Terminology Criteria for Adverse Events version 4.03.

^a^Neutropenia refers to the combined preferred terms of neutropenia and neutrophil count decreased.

^b^Leukopenia refers to the combined preferred terms of leukopenia and white blood cell count decreased.

^c^Thrombocytopenia refers to the combined preferred terms of thrombocytopenia and platelet count decreased.

To date for cohort 6, 24 patients (60%) had any-grade infections (11 [28%] Grade ≥3). Five patients (13%) had COVID-19 infections (3 [8%] Grade ≥3), none related to axi-cel treatment per investigator assessment (Table S3). One additional death due to COVID-19 was reported; the COVID-19 infection was not reported as a Grade 5 AE given that it occurred outside of the protocol-specified AE reporting period. Since the 1-year analysis [11], six new infections were reported, including COVID-19 (Grades 1, 2, and 5 [each *n* = 1]), Grade 3 *Pneumocystis jirovecii* pneumonia, Grade 3 unknown infectious episode with inflammatory syndrome, and Grade 2 herpes zoster. The latter three events were axi-cel–related per investigator assessment (Table S4). At month 3, the first assessment of B-cell levels post–axi-cel, 1/18 evaluable patients (6%) in ongoing response had detectable B cells. At 2 years, 5/16 evaluable patients (31%) had detectable B cells (Table S5). To date, 8 patients (20%) had hypogammaglobulinemia; all were Grade 1 (*n* = 2) or 2 (*n* = 6). Seven patients (18%) received intravenous immunoglobulin (IVIG) therapy per investigator’s discretion, and all uses were for AE treatment, although one patient also received IVIG for prophylaxis.

Since the 1-year analysis [11], the incidence of CRS was unchanged (Table 2; Supplementary results). No Grade ≥3 CRS events have occurred to date in cohort 6. Two new treatment-emergent NEs were observed in 2 patients since the 1-year analysis [11] (Table 2) and were ongoing at time of data cutoff (Table S6). One patient had Grade 2 dementia that was unrelated to axi-cel per investigator assessment (onset on day 685). The second patient had Grade 5 axi-cel–related (per investigator assessment) leukoencephalopathy that was ultimately fatal on day 815. A brain biopsy performed on day 802 suggested that the underlying etiology of the leukoencephalopathy was infection versus other malignancy. Serology testing of cerebrospinal fluid indicated the presence of antibodies to JC virus, suggesting the event may have been caused by JC virus. However, an autopsy was not performed. The patient was in CR at time of death and died in hospice care. Gamma globulin level was low at 238 mg/dL on day 734; the patient received chronic IVIG support until day 734. Since the Grade 5 event was coded as a NE, the incidence of Grade ≥3 NEs increased from 15% to 18% since the 1-year analysis.

**Table 2:** Summary of treatment-emergent CRS and neurologic events since start of study.

	Cohort 6 *N* = 40
CRS	
Any, *n* (%)	32 (80)
Worst Grade 1, *n* (%)	14 (35)
Worst Grade 2, *n* (%)	18 (45)
Worst Grade 3, *n* (%)	0
Worst Grade 4, *n* (%)	0
Worst Grade 5, *n* (%)	0
Worst Grade ≥3, *n* (%)	0
Median (range) time to onset of any-grade CRS, days	5 (1, 15)
Median (range) duration, days	4 (1, 11)
Patients for whom events resolved, *n/N* (%)	32/32 (100)
Neurologic events	
Any, *n* (%)	23 (58)
Worst Grade 1, *n* (%)	9 (23)
Worst Grade 2, *n* (%)	7 (18)
Worst Grade 3, *n* (%)	3 (8)
Worst Grade 4, *n* (%)	2 (5)
Worst Grade 5, *n* (%)	2 (5)
Worst Grade ≥3, *n* (%)	7 (18)
Median (range) time to onset of any-grade neurologic event, days	6 (2–162)
Median (range) duration, days	19 (1–438)
Patients for whom events resolved, *n/N* (%)	18/23 (78)

Severity of CRS was graded per modified Lee 2014 criteria [21]. Neurologic events were identified using a Medical Dictionary for Regulatory Activities version 24.1 search term list that was developed based on a modification of the specific search strategy by Topp et al [22]. The severity of neurologic events was graded with the use of the Common Terminology Criteria for Adverse Events, version 4.03, of the National Cancer Institute.

*CRS* cytokine release syndrome.

Eight deaths occurred since the 1-year analysis, including five due to progressive disease and three from aforementioned AEs (leukoencephalopathy [*n* = 1] and COVID-19 [*n* = 2]). No cases of replication-competent retroviruses or secondary malignancies have been reported thus far in cohort 6.

### Efficacy

The ORR was 95% (95% CI, 83–99%) and the CR rate was 80% (95% CI, 64–91%), both unchanged from the 1-year analysis [11]. Among patients who received corticosteroids for prophylaxis only (*n* = 15) versus for prophylaxis and toxicity management (*n* = 25), the ORR was 100% (95% CI, 78–100%) versus 92% (95% CI, 74–99%) and CR rates were 73% (95% CI, 45–92%) versus 84% (95% CI, 64–95%), respectively. As previously reported [10], median cumulative cortisone-equivalent corticosteroid doses were 1252 mg and 2504 mg among those who received corticosteroids for prophylaxis only versus prophylaxis and AE management, respectively.

**Fig. 1:**
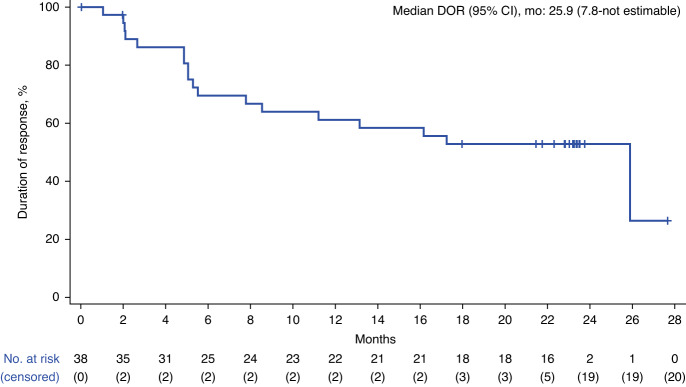
Duration of response. Disease assessment after initiation of new anticancer therapy (not including stem cell transplant) was not included in the duration of response derivation. CI, confidence interval; DOR, duration of response.

**Fig. 2:**
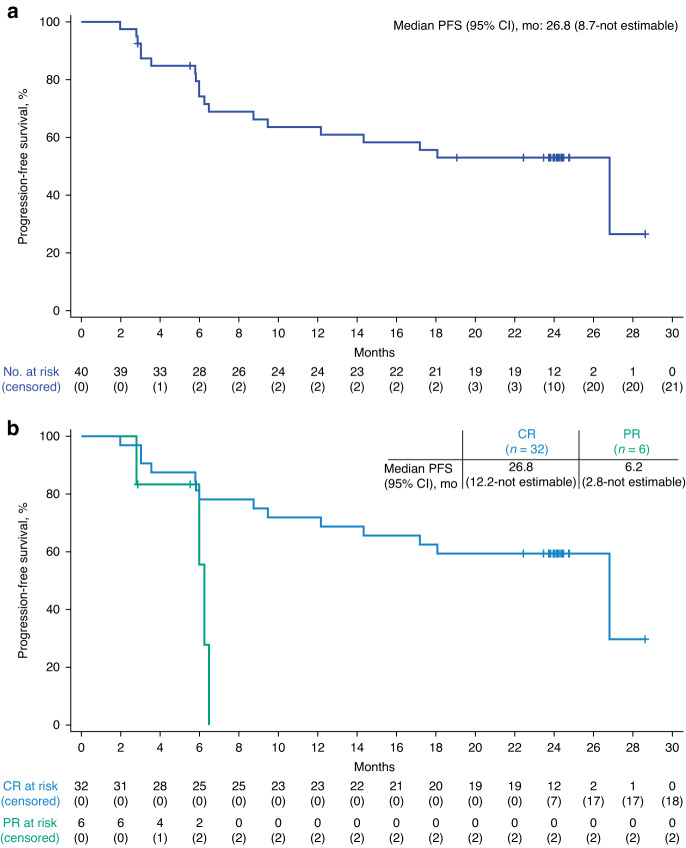
Progression-free survival. **a** Progression-free survival. **b** Progression-free survival by best overall response subgroup. Disease assessment after initiation of new anticancer therapy (not including stem cell transplant) was not included in the progression-free survival derivation. CI confidence interval, CR complete response, PFS progression-free survival, PR partial response.

**Fig. 3:**
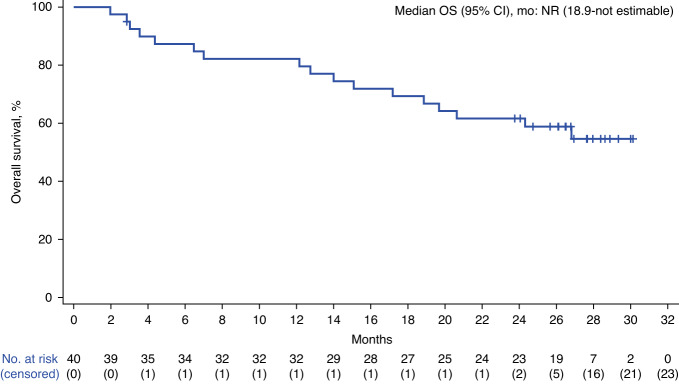
Overall survival. CI confidence interval, NR not reached, OS overall survival.

Since the 1-year analysis [11], one responder developed progressive disease and two responders died of AEs (patients’ last disease assessments were CR). Median DOR and PFS were reached at 25.9 months (95% CI, 7.8–not estimable) and 26.8 months (95% CI, 8.7–not estimable), respectively (Figs. 1–2). Median PFS in patients who achieved a best response of CR (*n* = 32) or PR (*n* = 6) was 26.8 months (95% CI, 12.2–not estimable) and 6.2 months (95% CI, 2.8–not estimable), respectively (Fig. 2b). Median OS was still not reached (95% CI, 18.9 months–not estimable; Fig. 3). Kaplan–Meier estimates of the 2-year DOR, PFS, and OS rates were 53% (95% CI, 36–68%), 53% (95% CI, 36–67%), and 62% (95% CI, 45–75%), respectively. Cumulative incidence rates of non–lymphoma-related mortality at 1 year and 2 years were 7.7% (95% CI, 1.9–18.9) and 15.4% (95% CI, 6.1–28.5), respectively. Of 18 patients (45%) in ongoing response at data cutoff, all achieved CR as best response.

Of the 40 patients in cohort 6, 32 were matched to those in cohorts 1 + 2 during PSM analysis. Eight patients from cohort 6 were not included due to nonavailability of matched patients in cohorts 1 + 2. Among the 32 matched patients in cohort 6 and cohorts 1 + 2, the 2-year cumulative incidence rate of lymphoma-related death was 26% (95% CI, 12–42%) and 25% (95% CI, 12–41%), respectively (Fig. S2) [10]. Among matched patients, non–lymphoma-related deaths were observed in 6 patients from cohort 6 and no patients in cohorts 1 + 2. Specifically, 2 patients in cohort 6 died of other reasons, and 4 died due to Grade 5 AEs (Table S7).

### Biomarker analyses

By month 24, 14/20 patients (70%) with evaluable blood samples had detectable levels of gene-marked anti-CD19 CAR T cells (Fig. S3A) versus 23/36 patients (64%) in cohorts 1 + 2. Similar to the 1-year analysis of cohort 6 [11], median peak CAR T-cell levels were higher in patients with ongoing response (61 cells/µl [*n* = 18]) or who relapsed by 2 years (68 cells/µl [*n* = 18]) versus nonresponders (18 cells/µl [*n* = 2]; Fig. S3B). A similar trend was observed with CAR T-cell expansion by AUC_0-28_. Notably, positive associations between median AUC_0-28_ and CRS severity, and median CAR T-cell peak and AUC_0-28_ with NE severity were observed (Supplementary results).

## Discussion

Based on the cohort 6 primary analysis [10], the United States Food and Drug Administration approved an update to the axi-cel prescribing information to include use of prophylactic corticosteroids for toxicity management across indications [2]. Moreover, the nursing guidelines on CAR T-cell therapy by the European Society for Bone Marrow Transplantation recommend the use of corticosteroids to manage therapy-related CRS and neurotoxicity [17]. With a median follow-up of 26.9 months, the cohort 6 findings reported herein demonstrate the long-term safety profile of axi-cel in R/R LBCL.

Similar to prior analyses [10, 11], the incidence, severity, and duration of CRS were decreased in cohort 6 versus cohorts 1 + 2, and the time to onset was delayed. Although 2 additional NEs were reported in 2 patients since the 1-year analysis for cohort 6 (one dementia and one infection as suspected etiologies) [11], the incidence and severity of NEs remain numerically lower than that reported in cohorts 1 + 2 [4, 10]. It should be noted that cohort 6 allowed for the use of bridging therapy after leukapheresis and before axi-cel, whereas cohorts 1 + 2 did not. Thus, it could be argued that the lower incidence of CRS and NEs in cohort 6 versus cohorts 1 + 2 could be attributable, in part, to better disease control prior to CAR T-cell therapy. However, median tumor burden (as assessed per sum of product diameters of target lesions) and lactate dehydrogenase levels were generally balanced in cohort 6 compared with cohorts 1 + 2 after PSM, where the differences in CRS and NEs remained apparent [18].

Beyond CRS and NEs, generally acute toxicities with early onset, cytopenia and immune deficiency are more frequently reported as late-onset AEs associated with CD19-targeted CAR T-cell therapy [19, 20]. Prolonged cytopenia is commonly reported, indicating a potential class effect [19]. B-cell aplasia represents an on-target/off-tumor effect of anti-CD19 CAR T-cell therapies, and patients may present with hypogammaglobulinemia, potentially requiring IVIG replacement to help mitigate infection risk [4, 19, 20]. Furthermore, corticosteroid use is independently associated with higher risk of infection [21, 22], which, when taken together, highlights infection as a key clinical consideration following CAR T-cell therapy. Incidence of Grade ≥3 prolonged cytopenia was numerically higher between patients in cohort 6 and cohorts 1 + 2 [4] in this unmatched analysis. However, B-cell recovery was observed over time in cohort 6 patients in ongoing response, similar to observations in cohorts 1 + 2 [4], and incidence of Grade ≥3 infections occurred at similar frequency between these patient groups [4]. These findings suggest that the cohort 6 toxicity management strategy did not lead to an added risk of infections versus that previously observed with the ZUMA-1 pivotal cohorts.

Clinically meaningful outcomes were observed in cohort 6 through ≥2 years of follow-up, and responses were consistent with prior analyses [10, 11]. Further, peak CAR T-cell levels were comparable in cohort 6 versus cohorts 1 + 2, suggesting no negative impact of corticosteroids on CAR T-cell pharmacokinetics [10]. A retrospective single-center experience with commercial axi-cel suggested that corticosteroid use may have prognostic impact, as higher cumulative doses (above the median cumulative dexamethasone-equivalent dose of 186 mg, *n* = 60) were associated with shorter PFS and OS, with no negative impact on CAR T-cell pharmacokinetics [23]. The median cumulative cortisone-equivalent corticosteroid dose including prophylaxis was 1252 mg for cohort 6 and 7418 mg for cohorts 1 + 2 [10], equal to 40 mg and 240 mg dexamethasone-equivalent doses, respectively. Although a greater proportion of patients in cohort 6 received corticosteroids compared with cohorts 1 + 2 (100% [as required per protocol] versus 22%, respectively) [10], patients in cohort 6 received less cumulative corticosteroids and median DOR and PFS were notably longer versus cohorts 1 + 2 [4]. Furthermore, the dexamethasone-equivalent corticosteroid dose in cohort 6 was less than one-third of the median dose reported in the aforementioned single-center experience [23], supporting the argument that prophylactic and earlier corticosteroid use may be associated with notably lower cumulative corticosteroid doses, and by that am improvement in the overall safety profile.

Median OS was not reached in cohort 6 or cohorts 1 + 2 [4] at 2 years, and lymphoma-related mortality was similar between these patient groups in this analysis (Fig. S2). These findings suggest that the cohort 6 toxicity management strategy did not increase likelihood of disease-related mortality. Indeed, all but 3 patients in cohort 6 who were in response at 1 year remained in response as of the 2-year data cutoff date, speaking to response durability [11]. Notably, non–lymphoma-related mortality was low in cohorts 1 + 2, with only 7 deaths reported among the 101 axi-cel–treated patients (3 deaths due to AEs and 4 for other reasons). None of these 7 patients were included in the PSM analysis set. Conversely, 6 patients in cohort 6 died of non–lymphoma-related reasons, and all were included in PSM. This difference may account for the lower overall mortality observed in cohorts 1 + 2 versus cohort 6 (Fig. S2).

Limitations of this study include the low number of patients enrolled, the lack of direct comparator arm, and those inherent with conducting an exploratory cohort study versus a randomized controlled trial. These factors may limit the interpretability of the results and, thus, additional confirmatory studies may be needed to further understand the impact of prophylactic corticosteroids on clinical practice and patient outcomes.

Collectively, with ≥2 years of follow-up, the ZUMA-1 cohort 6 toxicity management strategy continued to demonstrate reduced Grade ≥3 CRS without adversely affecting CAR T-cell pharmacokinetics or compromising efficacy outcomes, which remain high and durable, for patients with R/R LBCL treated with axi-cel.

### Supplementary information

Supplementary Appendix

## Data Availability

Kite is committed to sharing clinical trial data with external medical experts and scientific researchers in the interest of advancing public health, and access can be requested by contacting medinfo@kitepharma.com.
