# Supplementary material for: Long-term outcomes of patients with large B-cell lymphoma treated with axicabtagene ciloleucel and prophylactic corticosteroids
Source: Bone Marrow Transplant. 2024 Jan 4;59(3):366–72. doi: 10.1038/s41409-023-02169-z (PMC10920180; doi:10.1038/s41409-023-02169-z)
Supplement: Supplementary file 1 — Supplementary Appendix [file 41409_2023_2169_MOESM1_ESM.docx]

**Long-term outcomes of patients with large B-cell lymphoma treated with axicabtagene ciloleucel and prophylactic corticosteroids**

# SUPPLEMENTARY INFORMATION

Table of Contents

[Supplementary methods 2](#_Toc151034583)

[Study design 2](#_Toc151034584)

[Toxicity management strategy 2](#_Toc151034585)

[Coding and grading of adverse events 2](#_Toc151034586)

[Statistical analysis 2](#_Toc151034587)

[Supplementary results 4](#_Toc151034588)

[Safety 4](#_Toc151034589)

[Associations between CAR T-cell expansion and toxicity 4](#_Toc151034590)

[Propensity score matching analysis 4](#_Toc151034591)

[Table S1. Serious treatment-emergent adverse events reported in at least 2 patients since start of study. 6](#_Toc151034592)

[Table S2. Summary of cytopenias present on or after day 30 from axi-cel infusion since start of study. 7](#_Toc151034593)

[Table S3. Additional information on COVID-19 infections reported to date for cohort 6.* 8](#_Toc151034594)

[Table S4. Additional information on infections reported since the 1-year analysis.^6^ 9](#_Toc151034595)

[Table S5. Summary of B-cell aplasia among patients in ongoing response at 2 years. 10](#_Toc151034596)

[Table S6. Unresolved treatment-emergent neurologic events at time of data cutoff. 11](#_Toc151034597)

[Table S7. Non–lymphoma-related deaths in cohort 6 after propensity score matching. 12](#_Toc151034598)

[Figure S1. ZUMA-1 toxicity management strategy. 13](#_Toc151034599)

[Figure S2. Cumulative incidence of mortality among patients in cohort 6 and cohorts 1+2 after propensity score matching. 14](#_Toc151034600)

[Figure S3. (A) Anti-CD19 CAR T-cell levels over time through month 24 and (B) Associations between peak CAR T-cell levels and response at month 24. 15](#_Toc151034601)

[References 16](#_Toc151034602)

# Supplementary methods

## Study design

- The study protocol was approved by the institutional review board at each site. The study was conducted in accordance with the International Conference on Harmonization Good Clinical Practice guidelines, and all patients provided written informed consent
- Adults ≥18 years with histologically confirmed relapsed/refractory large B-cell lymphoma after ≥2 lines of systemic therapy and Eastern Cooperative Oncology Group performance status 0-1 were eligible. Patients were refractory to first-line therapy, or had progressive disease or relapsed within 12 months after autologous stem cell transplant, and must have received an anti-CD20 monoclonal antibody and an anthracycline-containing chemotherapy regimen

## Toxicity management strategy

- Specifically for cohort 6, Grade ≥1 neurologic events (NEs), Grade ≥2 cytokine release syndrome (CRS), and Grade 1 CRS with no improvement after 3 days were all managed with corticosteroids
- Grade ≥2 NEs with concurrent CRS, Grade ≥2 CRS, and Grade 1 CRS with no improvement after 24 hours were all managed with tocilizumab

## Coding and grading of adverse events

- Adverse events (AEs) were coded using Medical Dictionary for Regulatory Activities (MedDRA) version 24.1
  - Severity of AEs, excluding CRS, was graded using the National Cancer Institute Common Terminology Criteria for Adverse Events version 4.03

## Statistical analysis

- Safety was assessed in all treated patients (any axi-cel dose) and responses in the modified intent-to-treat population, which included all patients treated with the minimum or greater axi‑cel dose (≥1×10^6^ CAR T cells/kg)
- Time-to-event endpoints were analyzed using Kaplan-Meier method
- A post hoc analysis was performed to combine the following MedDRA preferred terms into 1 comprehensive term. As such, herein,
  - Neutropenia refers to the combined preferred terms of neutropenia and neutrophil count decreased
  - Leukopenia refers to the combined preferred terms of leukopenia and white blood cell count decreased
  - Thrombocytopenia refers to the combined preferred terms of thrombocytopenia and platelet count decreased

# Supplementary results

## Safety

The median time to onset of any-grade CRS was 5 days (range, 1-15), with a median duration of 4 days (range, 1–11). No changes in CRS symptoms were observed since the prior analysis. The most commonly reported signs and symptoms of CRS were pyrexia (97%), hypotension (53%), hypoxia (19%), and headache (16%).

Median time to onset of any-grade NE was 6 days (range, 2-162), with a median duration of 19 days (range, 1–438). The most frequent NEs of any grade were confusional state (38%), tremor (23%), aphasia (15%), and somnolence (15%).

## Associations between CAR T-cell expansion and toxicity

As no patients had Grade ≥3 CRS, associations between median peak CAR T‑cell levels and area under the curve within the first 28 days after treatment (AUC_0-28_) were compared between patients with Grade 2 (*n*=18) versus Grade 1 or no CRS (*n*=22). No association between median peak and CRS severity was noted between patients with Grade 2 versus Grade 1 or no CRS (84 cells/µl [range, 6–266] versus 60 cells/µl [range, 2–427]; *P*=0.06). Median AUC_0-28_ was 2.4-fold higher among patients with Grade 2 CRS (713 cells/µl [range, 74–3573]) versus Grade 1 or no CRS (294 cells/µl [range, 17–2712]; *P*=.04). With respect to NEs, median CAR T-cell peak was 2.0-fold higher among patients with Grade ≥3 events (*n*=7; 120 cells/µl [range, 30–172]) versus Grade ≤2 or no (*n*=33; 59 cells/µl [range, 2–427]) NEs (*P*=.03). Similarly, a positive association was observed with AUC_0-28_, with 3.0-fold higher AUC among patients with Grade ≥3 (1250 cells/µl [range, 461–3573]) versus Grade ≤2 or no (415 cells/µl [range, 17–2712]) NEs (*P*=.01).

## Propensity score matching analysis

ZUMA-1 safety management cohorts were exploratory and not powered to provide comparisons to pivotal cohorts 1+2.^1-3^ Without a randomized study, propensity score matching aims to minimize inherent differences between two distinct patient populations at baseline but cannot completely eliminate confounding effects of treatment on outcomes when using observational data.^4, 5^ Unidentified factors that are unaccounted for during cohort matching may still influence the analysis through residual confounding. Notably, these long-term cohort 6 findings are consistent with patterns observed previously and reported in the context of a propensity score matching analysis with ZUMA-1 pivotal cohorts 1+2.^3, 6^ However, the potential bias that may still exist with propensity score matching is a recognizable limitation of this methodology.

# Table S1. Serious treatment-emergent adverse events reported in at least 2 patients since start of study.

| ***n* (%)** | **Any grade** | **Worst Grade 3** | **Worst Grade 4** | **Worst Grade 5** |
| --- | --- | --- | --- | --- |
| **Any** | **24 (60)** | **10 (25)** | **3 (8)** | **7 (18)** |
| Confusional state | 5 (13) | 0 | 0 | 0 |
| Pyrexia | 4 (10) | 0 | 0 | 0 |
| Aphasia | 3 (8) | 0 | 0 | 0 |
| COVID-19 | 3 (8) | 1 (3) | 0 | 1 (3)* |
| Seizure | 3 (8) | 1 (3) | 2 (5) | 0 |
| Febrile neutropenia | 2 (5) | 1 (3) | 0 | 0 |
| Hypotension | 2 (5) | 1 (3) | 0 | 0 |
| Mental status changes | 2 (5) | 1 (3) | 1 (3) | 0 |
| Pancytopenia | 2 (5) | 1 (3) | 1 (3) | 0 |

Adverse events were coded using Medical Dictionary for Regulatory Activities version 24.1; severity was graded using the National Cancer Institute Common Terminology Criteria for Adverse Events version 4.03.

* One additional death due to COVID-19 was reported; the COVID-19 infection was not reported as a Grade 5 AE given that it occurred outside of the protocol-specified AE reporting period.

# Table S2. Summary of cytopenias present on or after day 30 from axi-cel infusion since start of study.

| ***n* (%)** | **Any grade** | **Worst Grade ≥3** |
| --- | --- | --- |
| **Any prolonged cytopenia** | **26 (65)** | **21 (53)** |
| **Prolonged thrombocytopenia** | **11 (28)** | **8 (20)** |
| Thrombocytopenia | 7 (18) | 5 (13) |
| Platelet count decreased | 4 (10) | 3 (8) |
| **Prolonged neutropenia** | **19 (48)** | **15 (38)** |
| Neutropenia | 12 (30) | 9 (23) |
| Neutrophil count decreased | 7 (18) | 6 (15) |
| Febrile neutropenia | 2 (5) | 1 (3) |
| **Prolonged anemia** | **7 (18)** | **4 (10)** |
| Anemia | 7 (18) | 4 (10) |

Axi-cel, axicabtagene ciloleucel; MedDRA, Medical Dictionary for Regulatory Activities; SMQ, Standardized MedDRA Query.

Adverse events were coded using MedDRA version 24.1; severity was graded using the National Cancer Institute Common Terminology Criteria for Adverse Events version 4.03. Prolonged thrombocytopenia/neutropenia/anemia were defined as thrombocytopenia/neutropenia/anemia present on or after 30 days from axi-cel infusion. Thrombocytopenia was identified using the SMQ hematopoietic thrombocytopenia (narrow). Neutropenia was identified using MedDRA search terms prespecified by the study sponsor: autoimmune neutropenia, band neutrophil count decreased, band neutrophil percentage decreased, febrile neutropenia, granulocyte count decreased, granulocytes abnormal, granulocytopenia, idiopathic neutropenia, mononuclear cell count decreased, neutropenia, neutropenic colitis, neutropenic infection, neutropenic sepsis, neutrophil count abnormal, neutrophil count decreased, neutrophil percentage abnormal, neutrophil percentage decreased, transfusion-related alloimmune neutropenia. Anemia was identified using the SMQ hematopoietic erythropenia (broad).

# Table S3. Additional information on COVID-19 infections reported to date for cohort 6.*

| **Patient** | **Preferred term** | **AE start date** | **AE end date** | **Toxicity grade** | **Causal relationship** | **SAE** | **Vaccination status** | **ANC** |
| --- | --- | --- | --- | --- | --- | --- | --- | --- |
| 1 | COVID-19 | 201 | 214 | 3 | None | Yes | N/A | Normal |
|  | COVID-19 | 276 | 331 | 1 | None | No |  |  |
| 2 | COVID-19 | 559 | 556 | 1 | None | No | N/A | Normal |
| 3 | COVID-19 | 473 | 480 | 4 | None | Yes | N/A | Normal* |
|  | COVID-19 | 481 | 507 | 2 | None | No |  |  |
|  | COVID-19 | 508 | 521 | 4 | None | Yes |  |  |
|  | COVID-19 | 522 | 522 | 5 | None | Yes |  |  |
| 4 | COVID-19 pneumonia | 213 | 226 | 3 | None | Yes | N/A | Normal |
| 5 | COVID-19 | 637 | 644 | 1 | None | No | Vaccinated (Pfizer) | N/A |
|  | COVID-19 | 645 | 649 | 2 | None | Yes |  |  |

AE, adverse event; ANC, absolute neutrophil count; N/A, not available; SAE, serious adverse event.

* One additional death due to COVID-19 was reported; the COVID-19 infection was not reported as a Grade 5 AE given that it occurred outside of the protocol-specified AE reporting period.

^†^ Normal 3 months prior to the first event.

# Table S4. Additional information on infections reported since the 1-year analysis.^6^

| **Patient** | **Preferred term** | **AE start date** | **AE end date** | **Toxicity grade** | **Causal relationship** | **SAE** | **IVIG** | **Prophylaxis** | **Concomitant medications for AE** |
| --- | --- | --- | --- | --- | --- | --- | --- | --- | --- |
| 6 | *Pneumocystis jirovecii*  pneumonia | 491 | 502 | 3 | Leukapheresis, conditioning chemotherapy, and axi-cel | Yes | No | Valaciclovir | Sulfamethoxazole,  trimethoprim, and  prednisone |
| 7 | Infection* | 453 | 476 | 3 | Axi-cel | No | No | Valaciclovir hydrochloride, sulfamethoxazole, trimethoprim, viral vaccines | N/A |
| 8 | Herpes zoster | 400 | 422 | 2 | Axi-cel | No | No† | Aciclovir and COVID-19 vaccine | Valaciclovir hydrochloride |

AE, adverse event; axi-cel, axicabtagene ciloleucel; IVIG, intravenous immunoglobulin; N/A, not available; SAE, serious adverse event.

* Unknown infectious episode with inflammatory syndrome.

† Patient was diagnosed with hypogammaglobulinemia the month prior to the event; no IVIG was given.

# Table S5. Summary of B-cell aplasia among patients in ongoing response at 2 years.

| ***n* (%)** | **Ongoing response *n*=18** |
| --- | --- |
| **B cells tested at baseline** | 18 (100) |
| No B cells | 10 (56) |
| With B cells | 8 (44) |
| **B cells tested at month 3** | 18 (100) |
| No B cells | 17 (94) |
| With B cells | 1 (6) |
| **B cells tested at month 6** | 14 (78) |
| No B cells | 11 (79) |
| With B cells | 3 (21) |
| **B cells tested at month 12** | 17 (94) |
| No B cells | 13 (76) |
| With B cells | 4 (24) |
| **B cells tested at month 15** | 16 (89) |
| No B cells | 11 (69) |
| With B cells | 5 (31) |
| **B cells tested at month 18** | 15 (83) |
| No B cells | 9 (60) |
| With B cells | 4 (40) |
| **B cells tested at year 2** | 16 (89) |
| No B cells | 11 (69) |
| With B cells | 5 (31) |

# Table S6. Unresolved treatment-emergent neurologic events at time of data cutoff.

| **Patient** | **Preferred term** | **AE start date** | **Duration as of data cutoff** | **Toxicity grade** | **Causal relationship** | **SAE** |
| --- | --- | --- | --- | --- | --- | --- |
| 10 | Leukoencephalopathy | 815 | 1 | 5 | Axi-cel | Yes |
| 11 | Dementia | 685 | 186 | 2 | None | No |
| 12 | Mental status changes | 76 | N/A* | 3 | None | Yes |
| 13 | Dementia | 93 | N/A† | 1 | None | No |
|  | Toxic encephalopathy | 369 | 1 | 5 | Axi-cel | Yes |
| 14 | Cognitive disorder | 48 | N/A^‡^ | 3 | None | Yes |

AE, adverse event; axi-cel, axicabtagene ciloleucel; N/A, not available; SAE, serious adverse event.

* Neurologic event was ongoing at time of death due to disease progression on Day 86, as previously reported.^3^

† Neurologic event was ongoing at time of death due to toxic encephalopathy on Day 369, as previously reported.^6^

^‡^ Neurologic event was ongoing at time of death due to urosepsis on Day 107, as previously reported.^3^

# Table S7. Non–lymphoma-related deaths in cohort 6 after propensity score matching.

| **Patient** | **Death date** | **Cause of death** | **Adverse event (if applicable)** |
| --- | --- | --- | --- |
| 1 | 18-Nov-2021 | Adverse event | Leukoencephalopathy |
| 14 | 18-Jan-2021 | Other | COVID-19 infection |
| 12 | 27-Sep-2020 | Other | Toxic encephalopathy |
| 13 | 29-Feb-2020 | Adverse event | Urosepsis |
| 15 | 10-Apr-2020 | Adverse event | Unknown* |
| 16 | 04-Mar-2020 | Adverse event | Respiratory failure |

* The patient died after end of study; thus, information on the adverse event that resulted in death is unknown.

# Figure S1. ZUMA-1 toxicity management strategy.

This figure was adapted and reproduced with permission.^3^

*Only in case of comorbidities or older age.

† Only if no improvement with tocilizumab; use standard dose.

^‡^ If no improvement after 24 hours of supportive care in cohort 6.

^§^ If no improvement after 3 days.

^||^ Only for Grade ≥2 NEs with concurrent CRS in cohort 6.

CRS, cytokine release syndrome; HD, high dose; NE, neurologic event.


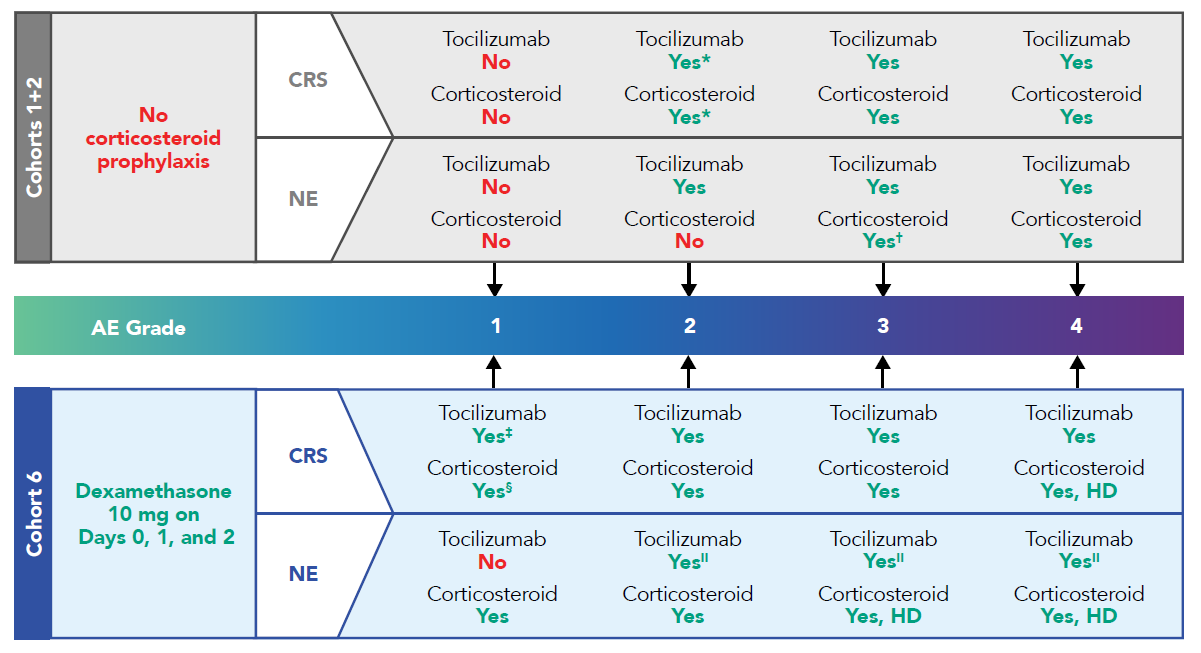


# Figure S2. Cumulative incidence of mortality among patients in cohort 6 and cohorts 1+2 after propensity score matching.

Eight patients from cohort 6 were not included due to nonavailability of matched patients in cohorts 1+2. OM and LSM are the same for cohorts 1+2, given that there are no patients among the matched set that died of non–lymphoma-specific risks.

CIF, cumulative incidence function; LSM, lymphoma-specific mortality; NLSM, non–lymphoma-specific mortality; OM, overall mortality.


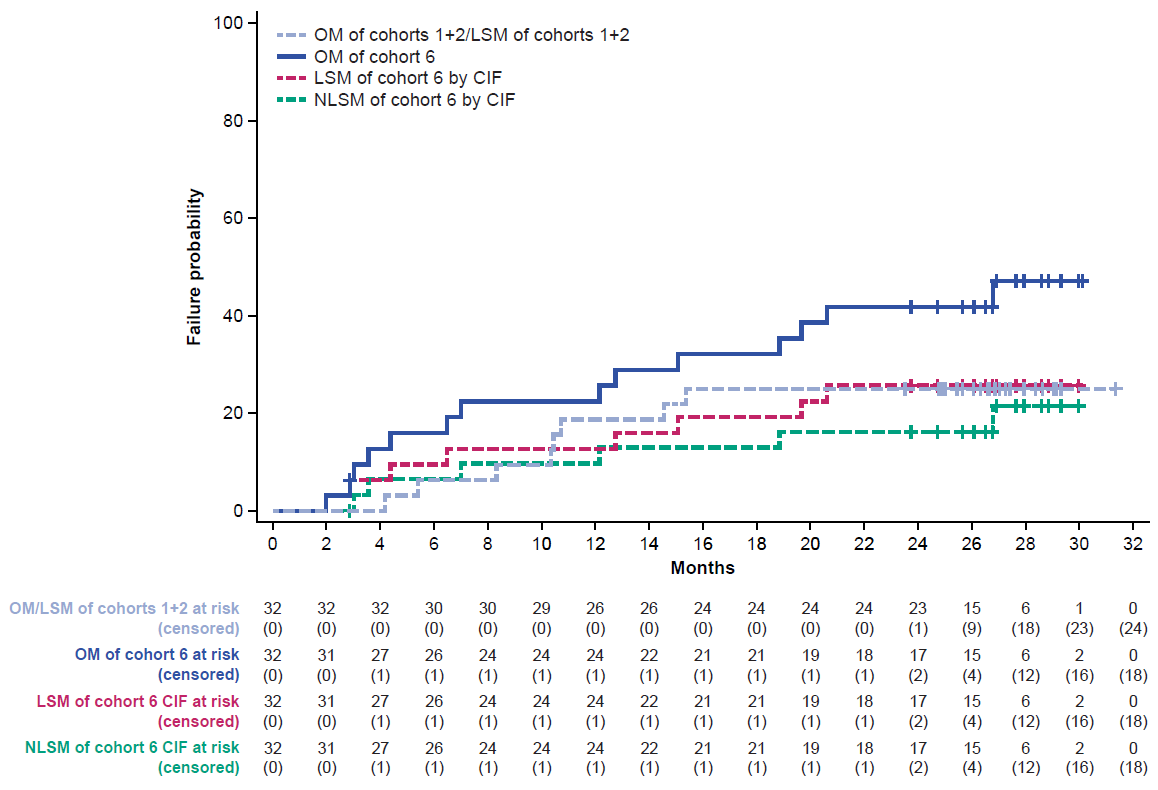


# Figure S3. (A) Anti-CD19 CAR T-cell levels over time through month 24 and (B) Associations between peak CAR T-cell levels and response at month 24.

For panel B, ongoing response was defined as patients who had complete or partial response (responders) by data cutoff. Relapse was defined as patients who responded (complete or partial response) but had documented progressive disease by time of data cutoff. Nonresponder was defined as patients who did not have either complete or partial response by the data cutoff date. Two patients who were responders (complete or partial response) at time of data cutoff were not included in the ongoing response assessment due to withdrawal of consent (*n*=1) and loss of follow-up (*n*=1).

CAR, chimeric antigen receptor.


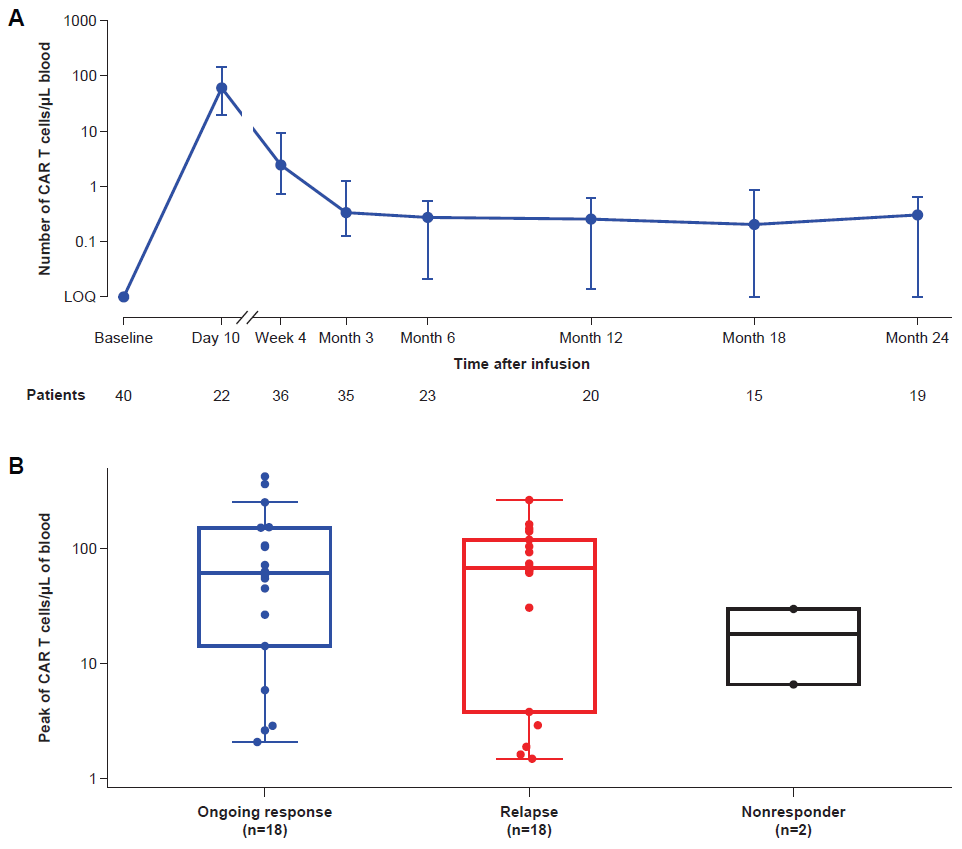


# References

1. Locke FL, Neelapu SS, Bartlett NL, Lekakis LJ, Jacobson CA, Braunschweig I et al. Preliminary results of prophylactic tocilizumab after axicabtagene ciloleucel (axi-cel; KTE-C19) treatment for patients with refractory, aggressive non-Hodgkin lymphoma (NHL). Blood 2017; 130(Suppl 1): 1547-1547.

2. Topp MS, van Meerten T, Houot R, Minnema MC, Bouabdallah K, Lugtenburg PJ et al. Earlier corticosteroid use for adverse event management in patients receiving axicabtagene ciloleucel for large B-cell lymphoma. Br J Haematol. 2021; 195(3): 388-398. doi: 10.1111/bjh.17673

3. Oluwole OO, Bouabdallah K, Munoz J, De Guibert S, Vose JM, Bartlett NL et al. Prophylactic corticosteroid use in patients receiving axicabtagene ciloleucel for large B-cell lymphoma. Br. J. Haematol. 2021; 194(4): 690-700. doi: 10.1111/bjh.17527

4. Rosenbaum PR, Rubin DB. The central role of the propensity score in observational studies for causal effects. Biometrika 1983; 70(1): 41-55.

5. Austin PC. An Introduction to propensity score methods for reducing the effects of confounding in observational studies. Multivariate Behav Res 2011; 46(3): 399-424. doi: 10.1080/00273171.2011.568786

6. Oluwole OO, Forcade E, Muñoz J, de Guibert S, Vose JM, Bartlett NL et al. Prophylactic corticosteroid use with axicabtagene ciloleucel (axi-cel) in patients (pts) with relapsed/refractory large B-cell lymphoma (R/R LBCL): One-year follow-up of ZUMA-1 Cohort 6 (C6). Blood 2021; 138: 2832. doi: <https://doi.org/10.1182/blood-2021-147403>
